# Creosote Collodion

**Published:** 1862-07

**Authors:** 


CREOSOTE COLLODION.—As an antisceptic, Mr. Stanislaus Martin employs a combination of fifteen parts by weight of creosote and ten of collodion. The solution is of a glutinous consistency.—*Am. Drug. Circular and Chem. Gaz.*

